# Supplementary figures and images for: Spectral-power associations reflect amplitude modulation and within-frequency interactions on the sub-second timescale and cross-frequency interactions on the seconds timescale
Source: PLoS One. 2020 May 18;15(5):e0228365. doi: 10.1371/journal.pone.0228365 (PMC7233599; doi:10.1371/journal.pone.0228365)

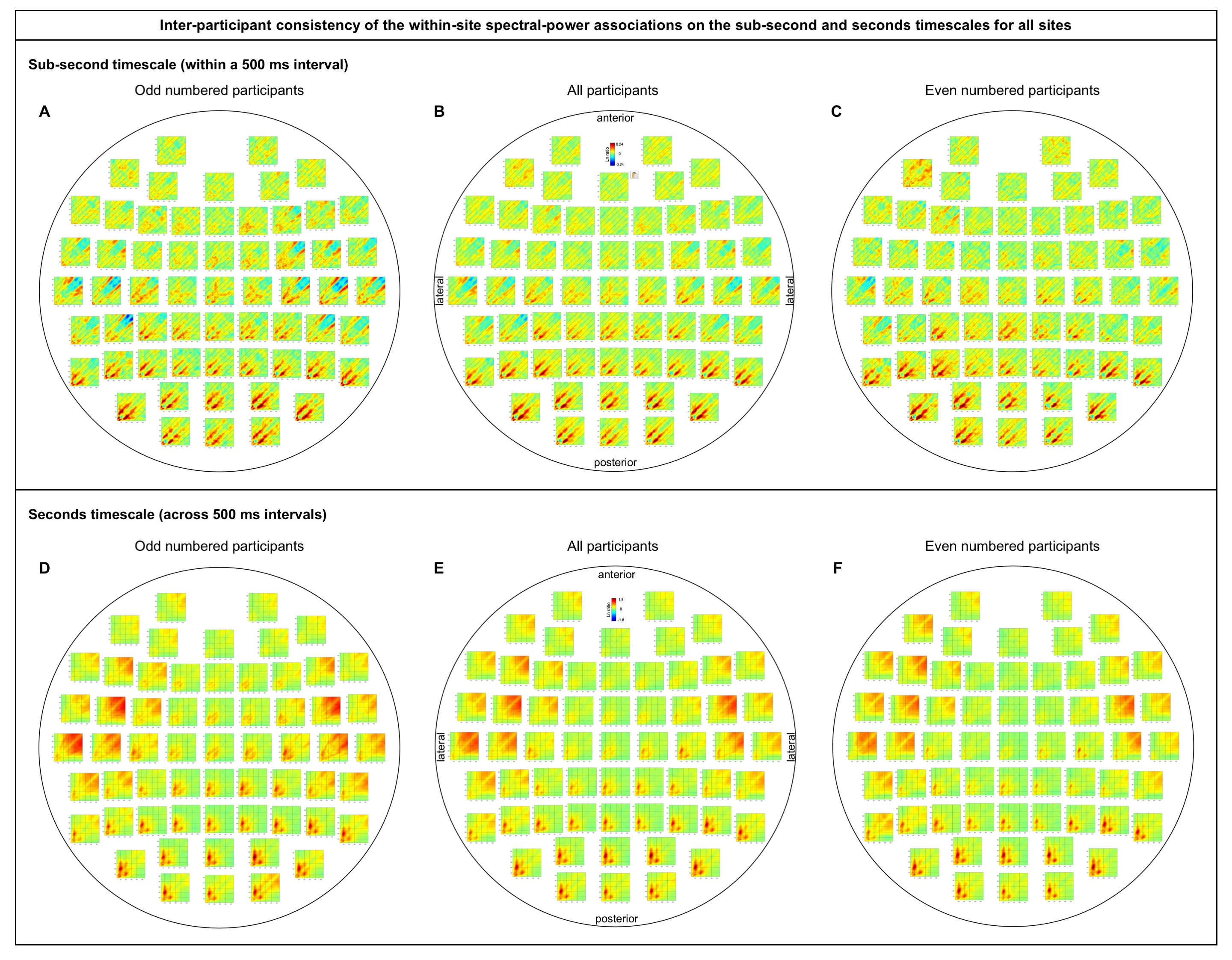

Supplement: S1 Fig — (JPEG) [file pone.0228365.s001.jpeg]

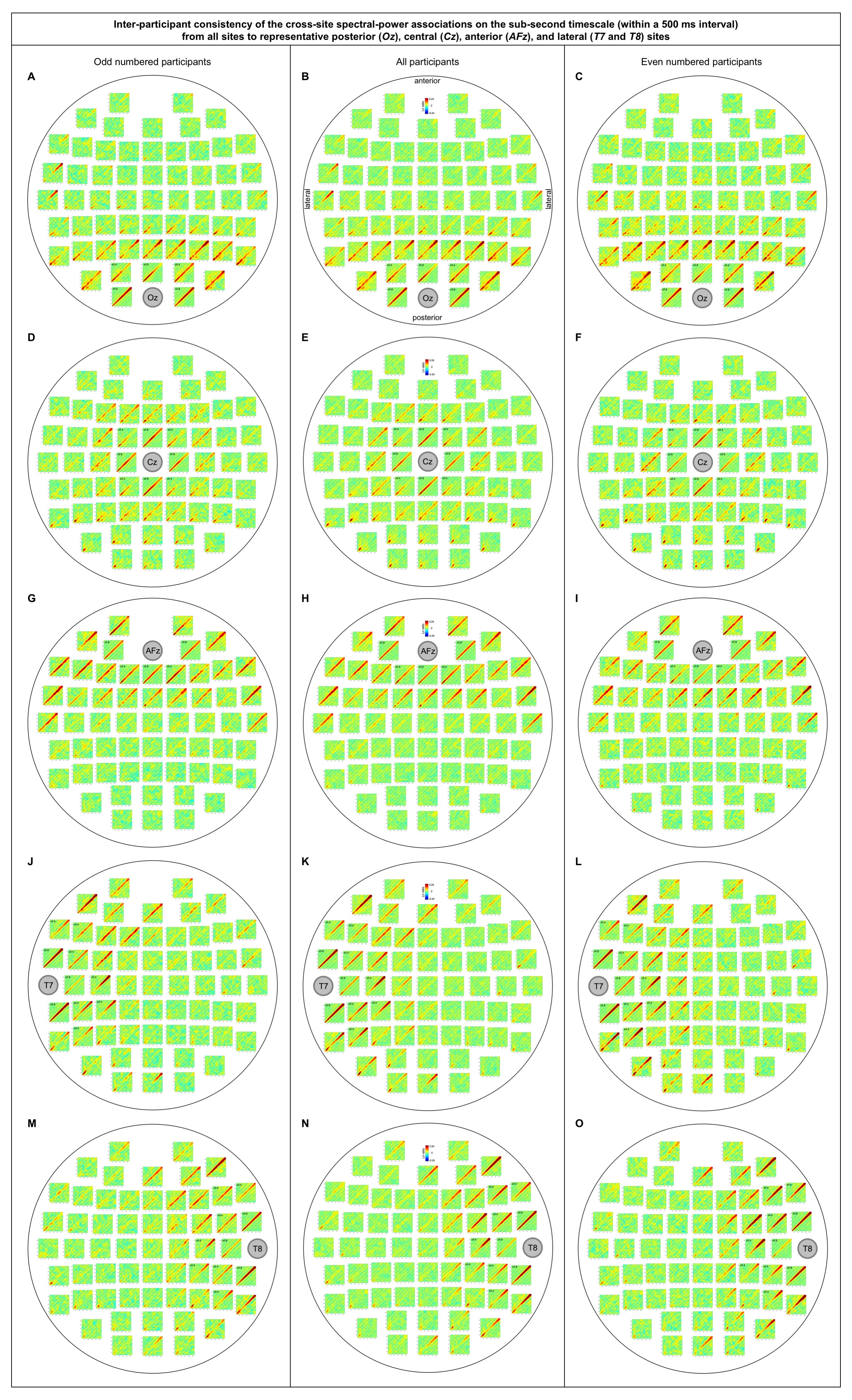

Supplement: S2 Fig — (JPEG) [file pone.0228365.s002.jpeg]

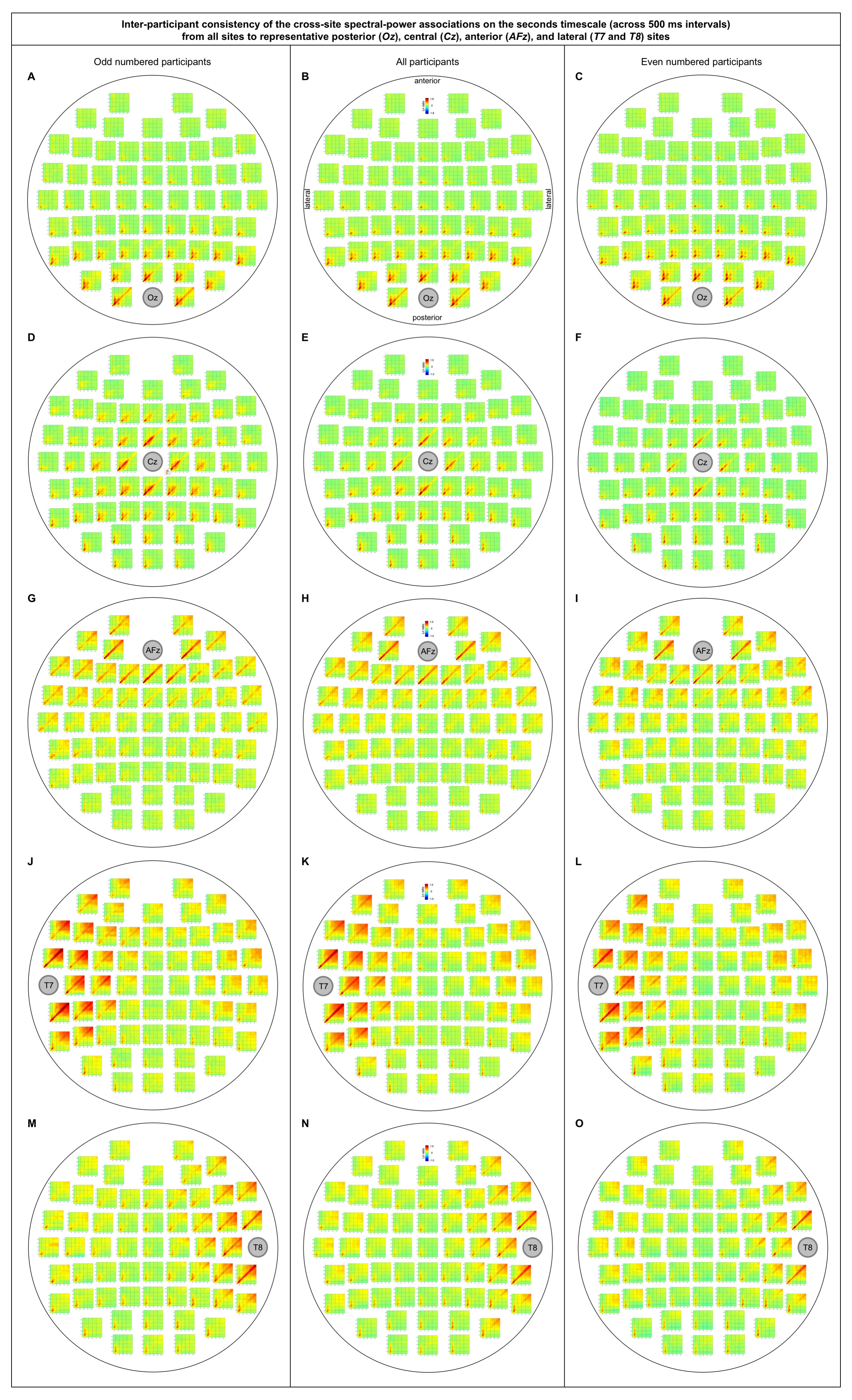

Supplement: S3 Fig — (JPEG) [file pone.0228365.s003.jpeg]
