# Supplementary material for: Spectral-power associations reflect amplitude modulation and within-frequency interactions on the sub-second timescale and cross-frequency interactions on the seconds timescale
Source: PLoS One. 2020 May 18;15(5):e0228365. doi: 10.1371/journal.pone.0228365 (PMC7233599; doi:10.1371/journal.pone.0228365)
Supplement: Attachment — Submitted filename: PONE-D-20-00985.pdf [file pone.0228365.s004.pdf]

Comments to the Author:

In this article, the authors present an original method to quantify spectral-power associations and used it to examine the intra- and inter-sites distribution of spectral-power associations in EEG recordings of subjects at rest. Two independent analysis were implemented for fast (sub-second) and slow (seconds) timescales. The authors also reflect about the relevance of their findings and provide some straightforward interpretations. The paper is therefore of relevance in the field of cross-frequency couplings and the analysis of its functional significance.

The experiments are well conceived and data analysis and statistics appears sound. I believe this paper will be of interest both to specialist in data processing and experimentalists. I would like to recommend the publication of the manuscript after the authors consider the revision of the following points.

(1) My first concern refers to the method used to mitigate the spurious positive spectral-power associations produced by partial wavelet overlap. Specifically, the authors propose to take the difference (in Ln-ratio) between the actual and DCT-phase-scrambled associations. I am a bit confusing about how this difference was implemented: between the actual association value and a single DCT-phase-scrambled association or the mean value of the distribution of the DCT-phase-scrambled associations? In any case, it would be better to compare the actual association value against the distribution of the DCT-phase-scrambled values. I think this is a relevant aspect of the data processing and should by clarified.

(2) In connection with the previous point, it is not clear for me how to interpret the negative values of the corrected spectral-power associations reported in the Probe frequency vs. Test frequency maps (e.g. Figure 3B in the manuscript). This negative associations are mentioned in the manuscript without providing any interpretation.

(3) I would like to suggest to the authors to consider using a traditional metric to quantify phase-amplitude coupling (e.g. Phase Locking Value, Modulation Index based on the Kullback-Leibler distance, See Tort 2010) on the analyzed EEG recordings to verify the presence of some phase-power cross-frequency couplings (CFCs) predicted in the study (e.g. Alpha vs. Gamma in the posterior region).

In my opinion, by incorporating this processing in the results (may be using a band-pass filtering technique not based in Morlet wavelets) would provide independent support to the findings and allow the readers to appreciate the potential of the proposed method to evaluate spectral-power associations as predictors of CFCs.

Tort, A.B.L., Komorowski, R., Eichenbaum, H., Kopell, N. (2010). Measuring Phase-Amplitude Coupling Between Neuronal Oscillations of Different Frequencies. Journal of Neurophysiology 104, 1195-1210. doi: 10.1152/jn.00106.2010.

(4) The analysis used to identify the fast (sub-second) and slow (seconds) timescales do not allow to discard the possibility of the presence of other intermediate characteristic timescales. I think, this should be discussed in the manuscript. Besides, Is the result shown in Fig. 2C robust against moderate increment of the time interval (e.g. 0,5sec., 1sec., 2sec.)?

(5) It is known that "spurious" phase-amplitude cross frequency coupling can be elicited due to the presence of spectral harmonics associated to the non sinusoidal "modulating" rhythm (Velarde 2019, Soldevilla 2016). It should be noted that the presence of harmonics associated to non sinusoidal low frequency rhythms constitutes a confounding factor that could also affect the assessment of spectral-power associations. For instance, the observed Alpha vs. Beta-Gamma spectral-power associations could reflect the presence of

spectral harmonics associated to the non sinusoidal oscillation in the Alpha-band, which extent inside the Beta and Gamma bands. In this scenario, the power fluctuations of the non sinusoidal rhythm in the Alpha band would produce the same power fluctuations in its spectral harmonics giving rise to spurious or epiphenomenal spectral-power associations in the sense that they reflect the interaction between dependent (harmonically related) spectral components rather than independent frequencies. This artifact could be particularly relevant in connection with the spectral-power associations observed in the posterior region since (non sinusoidal) oscillations in the Alpha-band are elevated in this region during the eyes closed state.
In my opinion, this is a relevant confounding factor related to the quantification of spectral-power associations that should be discussed in the manuscript.

Velarde, O.M., Urdapilleta, E., Mato, G., Dellavale, D. (2019). Bifurcation structure determines different phase-amplitude coupling patterns in the activity of biologically plausible neural networks. NeuroImage 202, 116031. doi: 10.1016/j.neuroimage.2019.116031.

Lozano-Soldevilla, D., ter Huurne, N., Oostenveld, R. (2016). Neuronal Oscillations with Non-sinusoidal Morphology Produce Spurious Phase-to-Amplitude Coupling and Directionality. Frontiers in Computational Neuroscience 10, 87. doi: 10.3389/fncom.2016.00087.

(6) I am not sure about point 3 on data availability.

(7) There are a few minor flaws and typos that should be fixed.

- In page 6: Where it says "discrete cosign transform" It shouls say "discrete cosine transform".

- In page 8: Where it says "f_{probet}-to-f_{test}" replace the subscript "probet" by "probe".

- In page 39: Where it says "being amplitude modulated at $|f_2-f_1|$ Hz" It shouls say "being amplitude modulated at $|f_2-f_1|/2$ Hz".
See for instance Background Theory Section and Figure 3 top-left in Berman 2012 and, Figure 2 in Aru 2015.

Berman, J.I., McDaniel, J., Liu, S., Cornew, L., Gaetz, W., Roberts, T.P., Edgar, J.C. (2012). Variable bandwidth filtering for improved sensitivity of cross-frequency coupling metrics. Brain Connectivity 2(3), 155-63. doi: 10.1089/brain.2012.0085.

Aru, J., Aru, J., Priesemann, V., Wibral, M., Lana, L., Pipa, G., Singer, W., Vicente, R. (2015). Untangling cross-frequency coupling in neuroscience. Curr. Opin. Neurobiol. 31, 51-61.
doi: 10.1016/j.conb.2014.08.002.
