# Supplementary material for: Spectral-power associations reflect amplitude modulation and within-frequency interactions on the sub-second timescale and cross-frequency interactions on the seconds timescale
Source: PLoS One. 2020 May 18;15(5):e0228365. doi: 10.1371/journal.pone.0228365 (PMC7233599; doi:10.1371/journal.pone.0228365)
Supplement: Attachment — Submitted filename: MencelogluEtAl_Response_to_Reviewers.pdf [file pone.0228365.s005.pdf]

Dear Dr. Vyazovskiy,

Thank you for obtaining the two thoughtful reviews and giving us the opportunity to revise the manuscript. We also thank both reviewers for reading through the long manuscript with numerous figures and making helpful and constructive comments. We very much appreciate their time and effort. Our responses to each of the reviewers' comments are presented below (blue text). We hope that you and the reviewers will find the revision to be acceptable. Thank you again for considering the manuscript for publication in *PLoS ONE*.

Sincerely,

Satoru Suzuki
satoru@northwestern.edu

Reviewer 1: Damián Dellavale

(1) My first concern refers to the method used to mitigate the spurious positive spectral-power associations produced by partial wavelet overlap. Specifically, the authors propose to take the difference (in Ln-ratio) between the actual and DCT-phase-scrambled associations. I am a bit confusing about how this difference was implemented: between the actual association value and a single DCT-phase-scrambled association or the mean value of the distribution of the DCT-phase-scrambled associations? In any case, it would be better to compare the actual association value against the distribution of the DCT-phase-scrambled values. I think this is a relevant aspect of the data processing and should by clarified.

The latter (the mean value of the distribution of the DCT-phase-scrambled associations). Control spectral-power association values (per site per participant) were computed based on four versions of phase-scrambled data and their mean values were subtracted (in Ln-ratio) from the real association values. The patterns of control association values were highly similar among the four phase-scrambled versions, so that the patterns of the control-subtracted spectral-power associations presented in the manuscript would appear virtually identical even if only one version of phase-scrambled data was used. We did not clearly explain this in the original manuscript and we thank the reviewer for pointing that out. We hope that the description is clear in the revision.

Relevant sections from the revised manuscript:

Page 6-7: "We used EEGd from 60 sites in conjunction with their DCT-phase-scrambled controls to extract intrinsic spectral-power associations over and above any artifact due to partial wavelet overlaps. Because of the 5 min length of the data (containing 153,600 time points at the 512 Hz sampling rate), 200 wavelet-center frequencies, and 60 sites per participant, even one set of phase-scrambled data would be unlikely to generate accidental patterns of spectral-power associations. We verified that the control patterns of spectral-power associations generated with different versions of phase-scrambled data (using different random seeds) were virtually indistinguishable, especially when two or more control patterns were averaged. To be conservative, we generated four versions of phase-scrambled data to compute four sets of control spectral-power associations (per site per participant), and used their averages as the controls (see below). We are thus reasonably certain that the control patterns accurately captured the spurious associations due to partial wavelet overlap."

(2) In connection with the previous point, it is not clear for me how to interpret the negative values of the corrected spectral-power associations reported in the Probe frequency vs. Test frequency maps (e.g. Figure 3B in the manuscript). This negative associations are mentioned in the manuscript without providing any interpretation.

We thank the reviewer for pointing this out. We did not clearly explain the interpretations of positive, negative, and near zero values of the spectral-power association values that we used. Positive association values indicate that the top/bottom-15% variation in the probe-frequency power was associated with greater same-signed modulation in the test-frequency power in Ln-ratio (analogous to positive correlation in that the test-frequency power was higher when the probe-frequency power was in the top 15% than when it was in the bottom 15%). Negative association values indicate that the top/bottom-15% variation in the probe-frequency power was associated with greater opposite-signed modulation in the test-frequency power in Ln-ratio (analogous to negative correlation in that the test frequency power was lower when the probe-frequency power was in the top 15% than when it was in the bottom 15%). Association values near zero indicate a lack of consistent associations (i.e., the top/bottom-15% power in the probe frequency tends to be coincident with higher or lower test-frequency power with equal probability). This expanded description of the spectral-power association values is included in the revision.

Relevant sections from the revised manuscript:

Page 8: "Thus, a larger positive association value indicates that the top/bottom-15% variation in the probe-frequency power was associated with greater *same-signed* co-variation in the test-frequency power in Ln-ratio (analogous to positive correlation, indicating that the test-frequency power was higher when the probe-frequency power was in the top 15% than when it was in the bottom 15%). A larger negative association value indicates that the top/bottom-15% variation in the probe-frequency power was associated with greater *opposite-signed* co-variation in the test-frequency power in Ln-ratio (analogous to negative correlation, indicating that the test frequency power was lower when the probe-frequency power was in the top 15% than when it was in the bottom 15%). Association values near zero indicate the lack of consistent association (i.e., the top/bottom-15% power in the probe frequency tended to be equivalently coincident with higher or lower test-frequency power)."

Page 9: "A larger positive association value indicates that the top/bottom-15% variation in the probe frequency power at *S1* was associated with greater *same-signed* co-variation in the test-frequency power at *S2* in Ln-ratio (analogous to positive correlation, indicating that the test-frequency power at *S2* was higher when the probe-frequency power at *S1* was in the top 15% than when it was in the bottom 15%). A larger negative association value indicates that the top/bottom-15% variation in the probe-frequency power at *S1* was associated with greater *opposite-signed* co-variation in the test-frequency power at *S2* in Ln-ratio (analogous to negative correlation, indicating that the test frequency power at *S2* was lower when the probe-frequency power at *S1* was in the top 15% than when it was in the bottom 15%). Association values near zero indicate the lack of consistent association (i.e., the top/bottom-15% power in the probe frequency at *S1* tended to be equivalently coincident with higher or lower test-frequency power at *S2*)."

In Figure 3 and elsewhere, we obtained negative associations on the fast (sub-second) timescale at $\Delta f \sim 8$ Hz for γ frequencies at lateral sites. This suggests that co-activation of γ frequencies at $\Delta f \sim 8$ Hz was actively reduced (relative to stochastic variations). We currently cannot offer any theoretical interpretation of this result.

(3) I would like to suggest to the authors to consider using a traditional metric to quantify phase-amplitude coupling (e.g. Phase Locking Value, Modulation Index based on the Kullback-Leibler distance, See Tort 2010) on the analyzed EEG recordings to verify the presence of some phase-power cross-frequency couplings (CFCs) predicted in the study (e.g. Alpha vs. Gamma in the posterior region). In my opinion, by incorporating this processing in the results (may be using a band-pass filtering technique not based in Morlet wavelets) would provide independent support to the findings and allow the readers to appreciate the potential of the proposed method to evaluate spectral-power associations as predictors of CFCs.

We thank the reviewer for making this suggestion. We used the measure of Modulation Index (Tort et al., 2010) to see whether we would observe the types of phase-amplitude coupling implied by the Δf associations we obtained on the sub-second timescale. We conducted this analysis for the Δf ~ 3 Hz associations for the θ-α frequencies and Δf ~ 10 Hz associations for the β-γ frequencies at posterior sites and the Δf ~ 16 Hz associations for the γ frequencies at lateral sites. In short, we did not find the implied phase-amplitude coupling for the Δf ~ 3 Hz associations, but we did confirm the implied phase-amplitude coupling for the Δf ~ 10 Hz and Δf ~ 16 Hz associations. Below is the relevant section in the revision.

Relevant sections from the revised manuscript:

Page 50-52: "So far, we have inferred occipital α-phase-to-β-γ-amplitude coupling based on the characteristic Δf ~ 10 Hz spectral-power associations for the β-γ frequencies on the sub-second timescale (e.g., Figure 3A). Additionally, we observed the Δf ~ 3 Hz spectral-power associations for the θ-α-frequencies at posterior sites (e.g., Figure 3A). Does this imply δ-phase-to-θ-α-amplitude coupling in the posterior region? We also observed the Δf ~ 16 Hz associations for the γ frequencies at lateral sites (e.g., Figure 3B). Does this imply lowβ-phase-to-γ-amplitude coupling in the lateral region?

We sought corroborating evidence for the relationship between the Δf associations and phase-amplitude coupling. Our strategy was to determine whether the types of phase-power coupling implied by the Δf spectral-power associations matched the phase-amplitude coupling assessed with a standard method of computing the Modulation Index, *MI* (e.g., Tort et al., 2010; Hülsemann et al., 2019). In short, the mean amplitude of the faster frequency band—amplitude frequency or *Af*—that coincides with each phase bin of the slower frequency band—phase frequency or *Pf*—is computed (over some temporal interval) to determine the degree to which the distribution of the *Af* amplitude over the *Pf* phase is peaky (i.e., the degree to which large *Af* amplitudes are concentrated at specific phase(s) of the *Pf* oscillation), using an entropy measure. The specific equation of *MI* yields a value of 0 if the distribution is flat and 1 if the distribution is maximally peaky (i.e., if the *Af* amplitudes are non-zero only at a single *Pf* phase bin). We used the full data length (~300 s) to compute *MI*'s, and also subtracted the control *MI*'s computed with the corresponding phase-scrambled data (per site per participant).

The sub-second Δf ~ 3 Hz associations for the θ-α-frequencies at posterior sites (e.g., Figure 3A) potentially imply a selective coupling of the θ-α amplitudes to the phase of the δ-band oscillations. However, we did not observe that the *MI*'s for the *Pf* of 3-4 Hz was selectively elevated for the *Af* of θ-α bands at the corresponding sites (data not shown). Thus, it is inconclusive as to whether the Δf ~ 3 Hz associations at posterior sites imply phase-amplitude coupling.

The results were more encouraging for the Δf ~ 10 Hz and Δf ~ 16 Hz associations. The Δf ~ 10 Hz associations for the β-γ-frequencies at posterior sites potentially imply a coupling of β-γ amplitudes to the phase of the α-band oscillations (a representative example from *PO8* shown in Figure 15B). This would selectively elevate *MI* for the combinations of *Pf* = 8-12 Hz (α band) with *Af* = 15-30 Hz (β band) and *Af* = 30-55 Hz (γ band). This prediction was confirmed (representative results from *PO8* shown in Figure 15C; see the data points highlighted in the dashed rectangle). To confirm selectivity, *MI* for the combination of *Pf* =13-17 Hz (lowβ band) with neither *Af* = 15-30 Hz (β band) nor *Af* = 30-55 Hz (γ band) was elevated (see non-highlighted data points in Figure 15C). Similarly, the Δf ~ 16 Hz associations for the γ-frequencies at lateral sites (e.g., Figure 3B) potentially imply a coupling of the γ amplitudes to the phase of the lowβ-band oscillations (a representative example from *C6* shown in Figure 15E). This would selectively elevate *MI* for the combination of *Pf* = 13-17 Hz (lowβ band) with *Af* = 30-55 Hz (γ band). This prediction was confirmed (representative results from *C6* shown in Figure 15F; see the data point highlighted in the dashed rectangle). To confirm selectivity, *MI* for the combination of *Pf* = 13-17 Hz (lowβ band) with *Af* = 15-30 Hz (β band) was not elevated; neither was *MI* elevated for the combination of *Pf* = 8-12 Hz (α band) with *Af* = 15-30 Hz (β band) or *Af* = 30-55 Hz (γ band) (see non-highlighted data points in Figure 15F).

We compared the spatial distributions of the Δf ~ 10 Hz and Δf ~ 16 Hz spectral-power associations (Figure 15A) with the spatial distributions of *MI* values for the expected phase-power coupling. To this end, we plotted the spatial distribution of the average of the *MI* values for the combinations of *Pf* = 8-12 Hz (α band) with *Af* = 15-30 Hz (β band) and *Af* = 30-55 Hz (γ band), predicted to be elevated in relation to the Δf ~ 10 Hz spectral-power associations (e.g., Figure 15B-C). These *MI* values were first *z*-normalized across sites per participant (*MIz*) so that the analysis focused on the spatial patterns of *MI*. The distribution of *MIz* covered the posterior region (Figure 15D, left) largely paralleling the posterior distribution of the Δf ~ 10 Hz association (Figure 15A). The posterior *MIz* distribution appears to be reasonably reliable as the patterns obtained from the odd and even numbered participants were similar (Figure 15D, right). Similarly, we plotted the spatial distribution of *MIz* for the combination of *Pf* = 13-17 Hz (low β band) with *Af* = 30-55 Hz (γ band), predicted to be elevated in relation to the Δf ~ 16 Hz spectral-power associations (e.g., Figure 15E-F). The distribution covered the lateral region (Figure 15G, left) largely paralleling the lateral distribution of the Δf ~ 16 Hz association (Figure 15A). Again, the lateral *MIz* distribution appears to be reasonably reliable as the patterns obtained from the odd and even numbered participants were similar (Figure 15G, right).

These results provide indirect evidence suggesting that at least the spectral-power associations at Δf ~ 10 Hz and Δf ~ 16 Hz on the sub-second timescale are related to amplitude-modulation of higher-frequency oscillations by the phase of Δf oscillations. For the posterior Δf ~ 10 Hz associations on the sub-second timescale that imply α-phase-to-β-γ-amplitude coupling, our results suggest a coherent interpretation relating them to the α-column associations on the seconds timescale observed at posterior sites and between posterior and non-posterior sites (see above). However, the current results do not provide a coherent interpretation for the lateral Δf ~ 16 Hz associations that imply lowβ-phase-to-γ-amplitude coupling."

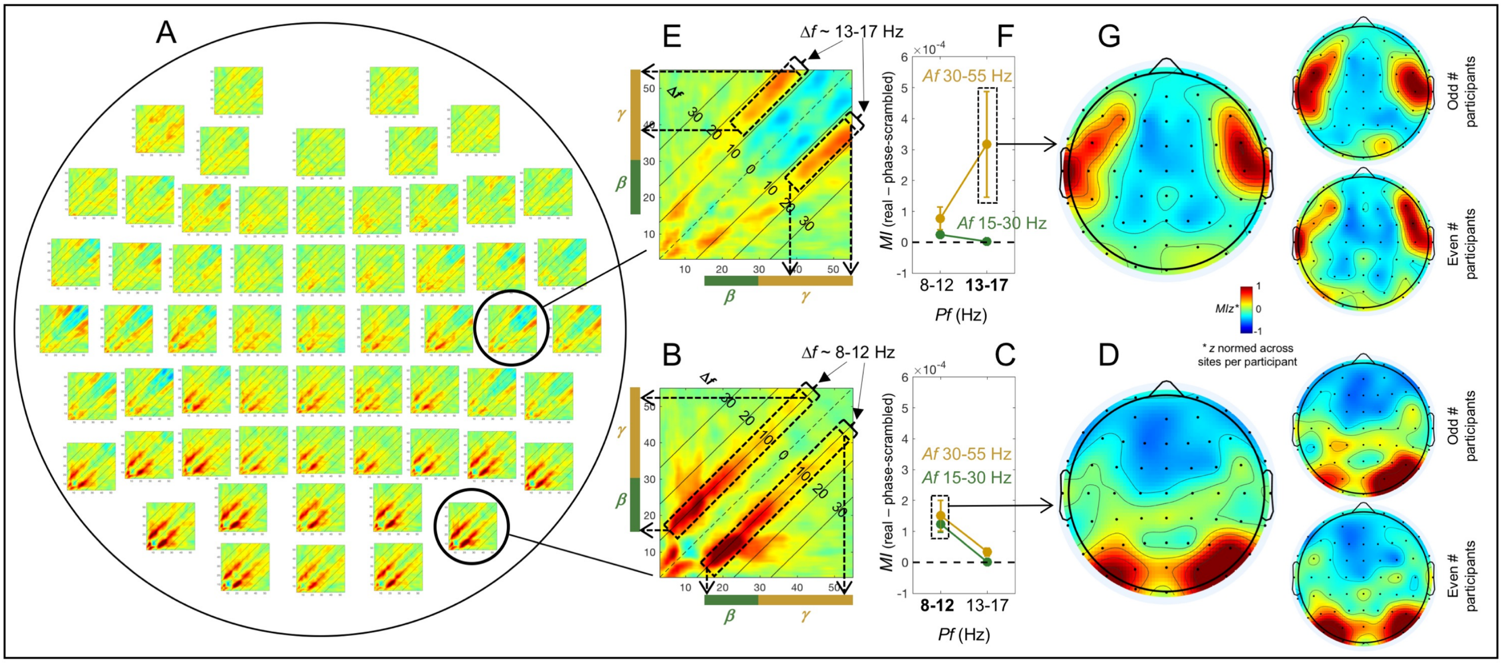

Figure 15. Relationship between the posterior Δf ~ 10 Hz and lateral Δf ~ 16 Hz spectral-power associations on the sub-second timescale and phase-amplitude coupling assessed as the Modulation Index (*MI*). A. The spatial distribution of the spectral-power associations on the sub-second timescale (same as Figure 3D). **B.** A representative example of the posterior Δf ~ 10 Hz (8-12 Hz, α band) associations for the β-γ frequencies at *PO8*. This pattern of spectral-power associations may imply a phase-amplitude coupling of the phase-frequency band (*Pf*) corresponding to Δf (8-12 Hz, α band) with the amplitude-frequency band (*Af*) in both the β (green bars) and γ (gold bars) ranges (see dashed rectangles and arrows). **C.** The phase-amplitude coupling assessed as *MI* (*MI* for real data minus *MI* for phase-scrambled data) at *PO8*. As implied by the spectral-power associations shown in B, *MI* was elevated selectively for the combination of *Pf* = 8-12 Hz (α band) with both *Af* = 15-30 Hz (β band) and *Af* = 30-55 Hz (γ band) (highlighted with the dashed rectangle). To confirm selectivity, *MI* was not elevated for the combination of *Pf* = 13-17 (lowβ band) with *Af* = 15-30 Hz (β band) or *Af* = 30-55 Hz (γ band). The error bars represent ±1 standard error of the mean with participants as the random effect. **D. Left.** Spatial distribution of *MIz* (*z*-normalized across sites per participant to focus on the spatial patterns of *MI*) averaged for the combinations of *Pf* = 8-12 Hz (α band) with *Af* = 15-30 Hz (β band) and *Af* = 30-55 Hz (γ band) (e.g., the dashed rectangle in C) predicted to be elevated by the posterior spectral-power associations shown in B. The posterior distribution of *MIz* largely parallels the posterior distribution of the Δf ~ 8-12 Hz associations (see A). **Right.** The *MIz* distributions plotted separately for the odd and even numbered participants showing the degree of inter-participant variability. **E.** A representative example of the lateral Δf ~ 16 Hz (13-17 Hz, lowβ band) associations for the γ frequencies at *C6*. This pattern of spectral-power associations may imply a phase-amplitude coupling of the phase-frequency band (*Pf*) corresponding to Δf (13-17 Hz, lowβ band) with the amplitude-frequency band (*Af*) in the γ (gold bars) range (see the dashed rectangles and arrows). **F.** The phase-amplitude coupling assessed as *MI* (*MI* for real data minus *MI* for phase-scrambled data) at *C6*. As implied by the spectral-power associations shown in E, *MI* was elevated selectively for the combination of *Pf* = 13-17 Hz (lowβ band) with *Af* = 30-55 Hz (γ band) (highlighted with dashed rectangle). To confirm selectivity, *MI* was not elevated for the combination of *Pf* = 13-17 (lowβ band) with *Af* = 15-30 Hz (β band); neither was it elevated for the combination of *Pf* = 8-12 Hz (α band) with *Af* = 15-30 Hz (β band) or *Af* = 30-55 Hz (γ band). The error bars represent ±1 standard error of the mean with participants as the random effect. **G. Left.** Spatial distribution of *MIz* (*z*-normalized across sites per participant to focus on the spatial patterns of *MI*) for the combination of *Pf* = 13-17 Hz (lowβ band) with *Af* = 30-55 Hz (γ band) (e.g., the dashed rectangle in F) predicted to be elevated by the lateral spectral-power associations shown in E. The lateral distribution of *MIz* largely parallels the lateral distribution of the Δf ~ 13-17 Hz associations (see A). **Right.** The *MIz* distributions plotted separately for the odd and even numbered participants showing the degree of inter-participant variability.

(4) The analysis used to identify the fast (sub-second) and slow (seconds) timescales do not allow to discard the possibility of the presence of other intermediate characteristic timescales. I think, this should be discussed in the manuscript. Besides, Is the result shown in Fig. 2C robust against moderate increment of the time interval (e.g. 0,5sec., 1sec., 2sec.)?

This is a very good question. The reviewer is correct in that both the mean within-interval and across-interval top/bottom-state duration computed in the way described in our article depends on the duration of the analysis intervals. We have added the following justification for choosing the analysis intervals of 500 ms.

Relevant sections from the revised manuscript:

Page 12-13: "Note that temporal estimates of the fast and slow fluctuations depend on the choice of the analysis interval. We chose 500 ms partly because it is a "landmark" timescale for human perception, cognition, and action (see above). The use of 500 ms analysis intervals also facilitated the goal of identifying distinctly timescale-dependent spectral-power associations.

Spectral-power fluctuations include both fast and slow components as shown in Figure 2A and 2B. Thus, estimates of within-interval T/B-state durations would necessarily increase with the use of longer analysis intervals because slower fluctuations would be included in the estimates. Indeed, the mean within-interval T/B-state duration approximately logarithmically increased with the length of analysis intervals (Figure 2D). The mean across-interval T/B state duration increased approximately linearly with the length of analysis intervals (Figure 2F). This is not surprising because we used non-overlapping intervals so that the within- and across-interval analyses of spectral-power dynamics were mathematically independent. The use of non-overlapping intervals made the temporal resolution for measuring across-interval power fluctuations inversely related to the length of the analysis interval.

Interestingly, both the within- and across-interval T/B-state durations were similar for the real (black curves) and phase-scrambled (blue curves) data for short (< 1000 ms) analysis intervals (Figure 2D and 2F), suggesting that the fast and slow spectral-power fluctuations revealed with short (< 1000 ms) analysis intervals primarily depend on spectral-amplitude compositions regardless of phase relations. It is also noteworthy that inter-participant variability was low (thin lines in Figure 2D and 2F) for both the fast (within-interval) and slow (across-interval) T/B-state durations obtained with short (< 1000 ms) analysis intervals, especially for those obtained with 500 ms intervals.

As shown below, the use of 500 ms analysis intervals revealed robust patterns of fast (within-interval) spectral-power associations. For the purpose of characterizing the timescale dependence of spectral-power associations, the use of the shortest analysis interval that generates distinct patterns of spectral-power associations would be advantageous for characterizing spectral-power associations on the fastest timescale because the use of longer intervals would intermix slower associations. For characterizing distinct spectral-power associations operating on the slower (across-interval) timescale that are mathematically independent of the fast (within-interval) associations, the use of 500 ms analysis intervals (relative to the use of longer analysis intervals) would also be most effective by providing the highest temporal resolution for characterizing the slow across-interval dynamics. We verified this line of reasoning as follows.

We computed both within-interval and across-interval spectral-power associations for representative sites (for examining within-site spectral-power associations) and for representative site-pairs (for examining cross-site spectral-power associations), using 500, 1000, 2000, 4000, and 8000 ms analysis intervals. As shown below, the use of 500 ms analysis intervals revealed distinct patterns of fast (within-interval) and slow (across-interval) spectral power associations. Within-interval spectral-power associations computed with longer analysis intervals became progressively similar to the across-interval associations computed with a 500 ms analysis interval, confirming that the use of longer analysis intervals results in intermixing fast and slow associations. Across-interval spectral-power associations computed with longer analysis intervals largely maintained the patterns obtained with 500 ms analysis intervals except that the association magnitudes became weaker, confirming that the characteristic slow (across-interval) spectral-power associations are most effectively revealed with 500 ms analysis intervals (relative to longer analysis intervals).

In summary, the analyses presented in Figure 2 and the above observations suggest that the use of 500 ms analysis intervals reveals mathematically independent spectral-power dynamics on the sub-second (with high/low-power states typically lasting ~230 ms) and seconds (with high/low-power states typically lasting ~3.75 s) timescales with the following advantages: (1) these sub-second and seconds timescales are relatively stable, depending primarily on spectral-amplitude compositions regardless of phase relations, (2) they are highly consistent across participants, and most significantly (3) they appear to support maximally distinct patterns of spectral-power associations."

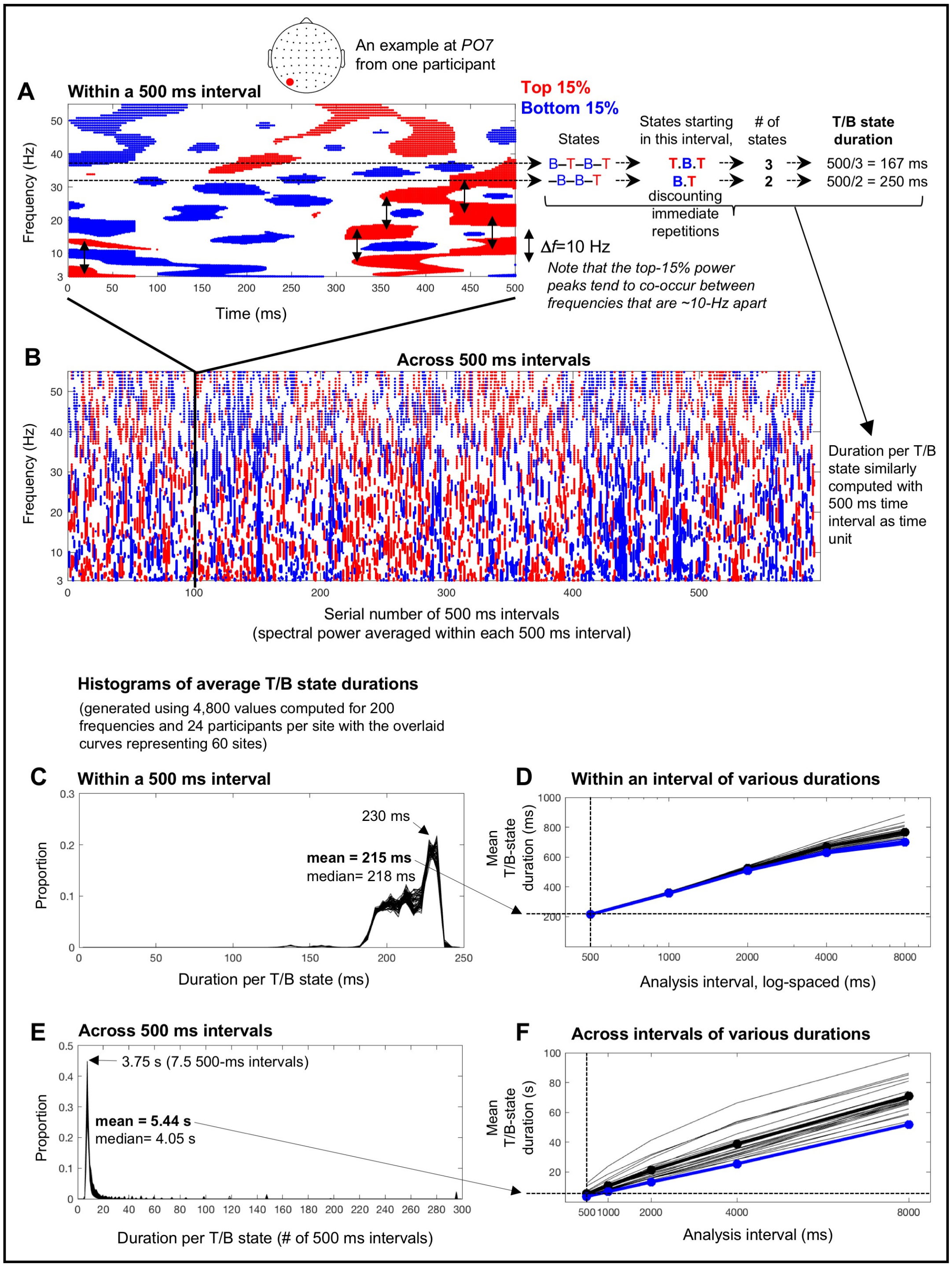

Figure 2. Intrinsic spectral-power variations on the characteristic sub-second (~230 ms) and seconds (~3.75 s) timescales. A. An example of the top-15% (red) and bottom-15% (blue) spectral-power variations within a 500 ms interval (taken from a 5 min EEG recording; see B) at a posterior site, *PO7*, from one participant. Note that spectral power tends to peak in pairs of frequencies that are ~10 Hz apart (the vertical arrows indicating $\Delta f \sim 10$ Hz). The average duration of the top and bottom states was estimated per frequency by identifying the state sequence ("B" for bottom, "T" for top, and "**–**" for in-between states in the illustration), counting the top and bottom states that started within the interval while discounting immediate repetitions, and dividing the interval duration (500 ms in this case) by the number of T/B states. Two examples are shown for two different frequencies (see text for details). The average T/B-state duration computed for each of the six-hundred 500 ms intervals over the 5 min EEG recording period were averaged per frequency per participant per site. **B.** An example of the top-15% (red) and bottom-15% (blue) power variations occurring across 500 ms intervals (spectral power averaged within each 500 ms interval) over the 5 min period. The average duration of the top and bottom states was estimated in the same way as for the 500 ms interval (see A) but using 500 ms intervals as time unit. **C.** Histograms of average within-500-ms-interval T/B-state durations (averaged across 600 intervals) based on the 4,800 values computed for 200 wavelet-center frequencies and 24 participants per site; histograms from the 60 sites are overlaid. The mode of ~230 ms with the mean of ~215 ms indicate that each brief high/low-power state typically lasted about a quarter of a second. **D.** Mean within-interval T/B-state durations similarly computed using different analysis intervals, 1000, 2000, 4000, and 8000 ms in addition to 500 ms, for both the real (black) and phase-scrambled (blue) data (the thin lines showing individual participants' data). The mean within-interval T/B-state duration approximately logarithmically increased with the length of analysis intervals. Note that the real and phase-scrambled curves virtually overlap, indicating that within-interval T/B-state durations depend primarily on spectral-amplitude compositions regardless of phase relations. Also note that individual differences are negligible for within-interval T/B-state durations computed with short (< 1000 ms) analysis intervals. **E.** Histograms of average across-500-ms-interval T/B-state durations based on the 4,800 values computed for 200 wavelet-center frequencies and 24 participants per site; histograms from the 60 sites are overlaid. The mode of ~3.75 s and the mean of 5.44 s indicate that each longer high/low-power state typically lasted several seconds. **F.** Mean across-interval T/B-state durations similarly computed using different analysis intervals (1000, 2000, 4000, and 8000 ms in addition to 500 ms) for both the real (black) and phase-scrambled (blue) data (the thin lines showing individual participants' data). The mean across-interval T/B-state duration approximately linearly increased with the length of analysis intervals. Note that the real and phase-scrambled curves nearly overlap for the 500 ms interval, indicating that T/B-state durations measured across 500 ms intervals primarily depend on spectral-amplitude compositions regardless of phase relations. Also note that individual differences are small for across-interval T/B-state durations computed with short (< 1000 ms) analysis intervals. We chose 500 ms as the analysis interval because it revealed the sub-second (within-interval) and seconds (across-interval) timescales of spectral-power fluctuations (1) that were relatively stable, depending on spectral-amplitude compositions regardless of phase relations, (2) that were highly consistent across participants, and (3) that appeared to support maximally distinct patterns of fast and slow spectral-power associations (see text for details).

(5) It is known that "spurious" phase-amplitude cross frequency coupling can be elicited due to the presence of spectral harmonics associated to the non sinusoidal "modulating" rhythm (Velarde 2019, Soldevilla 2016). It should be noted that the presence of harmonics associated to non sinusoidal low frequency rhythms constitutes a confounding factor that could also affect the assessment of spectral-power associations. For instance, the observed Alpha vs. Beta-Gamma spectral-power associations could reflect the presence of spectral harmonics associated to the non sinusoidal oscillation in the Alpha-band, which extent inside the Beta and Gamma bands. In this scenario, the power fluctuations of the non sinusoidal rhythm in the Alpha band would produce the same power fluctuations in its spectral harmonics giving rise to spurious or epiphenomenal spectral-power associations in the sense that they reflect the interaction between dependent (harmonically related) spectral components rather than independent frequencies. This artifact could be particularly relevant in connection with the spectral-power associations observed in the posterior region since (non sinusoidal) oscillations in the Alpha-band are elevated in this region during the eyes closed state. In my opinion, this is a relevant confounding factor related to the quantification of spectral-power associations that should be discussed in the manuscript.

We appreciate this suggestion and we now cite these relevant studies. We now include the following paragraph in the general discussion section.

Relevant sections from the revised manuscript:

Page 54: "When analyzing cross-frequency associations using frequency decomposition methods, one needs to be mindful of the artifactual harmonics generated by non-sinusoidal waveforms. In particular, recent computational studies have demonstrated that non-sinusoidal waveforms of α oscillations could generate harmonics that mimic the coupled β-γ oscillations (e.g., Lozano-Soldevilla, et al., 2016; Velarde, et al., 2019). This waveform artifact could contribute broad spectral-power associations among the α through β-γ frequencies as the power of artifactual β-γ harmonics would synchronously covary with the power of the non-sinusoidal α oscillations. Nevertheless, because the signals at each EEG current-source (site) reflect extensive spatial summation of neuronal signals at variable delays, any observable effects of artifactual spectral-power associations due to the harmonics generated by non-sinusoidal waveforms would have been attenuated (Schaworonkow & Nikulin, 2019). Further, most of the characteristic spectral-power associations we obtained on the sub-second and seconds timescales are not characterized by broad associations among the α through β-γ frequencies."

(6) I am not sure about point 3 on data availability.

We will upload all relevant data if/when the manuscript is accepted for publication.

(7) There are a few minor flaws and typos that should be fixed.

- In page 6: Where it says "discrete cosign transform" It should say "discrete cosine transform".

Fixed. We thank the reviewer for detecting this typo.

- In page 8: Where it says "fprobet-to-ftest" replace the subscript "probet" by "probe".

Fixed. We thank the reviewer for detecting this typo.

- In page 39: Where it says "being amplitude modulated at |f2-f1| Hz" It should say "being amplitude modulated at |f2-f1|/2 Hz". See for instance Background Theory Section and Figure 3 top-left in Berman 2012 and, Figure 2 in Aru 2015.

We carefully read the two suggested articles, but we are still uncertain as to why the amplitude modulation (beat) frequency for concurrent f_1 and f_2 oscillations should be $|f_2–f_1|/2$ instead of $|f_2–f_1|$.

We have,

$$\cos(2\pi f_1 t + \varphi_1) + \cos(2\pi f_2 t + \varphi_2) = 2\cos\left(2\pi\left[\frac{f_1+f_2}{2}\right] + \frac{\varphi_1+\varphi_2}{2}\right)\cos\left(2\pi\left[\frac{|f_1-f_2|}{2}\right] + \frac{\varphi_1-\varphi_2}{2}\right)$$

from the trigonometric identity, so that the sum exhibits the faster carrier oscillation at $\frac{f_1+f_2}{2}$ being multiplicatively modulated by the slower oscillation at $\frac{|f_1-f_2|}{2}$. However, because the amplitude modulation of the faster carrier-frequency oscillation does not depend on the sign of the slower multiplicatively-modulating factor, the frequency of amplitude modulation is $\frac{|f_1-f_2|}{2}\times 2$ or $|f_1 - f_2|$. It is likely that we are misunderstanding the reviewer's point or overlooking something; we would appreciate it if the reviewer could explain how our reasoning is faulty.

Reviewer 2

Reviewer #2: In this manuscript, the amplitude modulation and within-frequency interactions on two specific timescales of sub-second and seconds are illustrated using the plots of spectral-power associations. This approach is interesting to me. There are some comments for authors:

Major comments:
1) This approach focuses on the methodology to represent the spectral-power association among the recordings of multi-channels current source density in resting eye-close state. Any new findings with clear physiological meanings? There is no conclusion in this manuscript. Conclusions are important for audiences to catch your contributions and finding in this study.

We understand the reviewer's suggestion that we should discuss physiological implications of our results. However, additional experiments would be necessary—especially the application of the current spectral-power analyses to EEG data on various behavioral conditions as suggested by the reviewer in comment #4—to figure out the physiological and functional roles of the intrinsic spectral-power association patterns that we identified in the current study using rest-with-the-eyes-closed EEG. We understand the reviewer's dissatisfaction. We are currently applying the analyses to various behavioral conditions in a systematic way (and have applied for grant support) to understand how the observed intrinsic association patterns adjust (or stay invariant) to perceptual, attentional, and cognitive demands. Nevertheless, we feel that the current set of results characterizing intrinsic spectral-power associations merit publication in *PLoS ONE* even before our (and others') future results reveal their physiological and functional implications. Our understanding is that *PLoS ONE* focuses on disseminating rigorous and reliable scientific findings even prior to fully understanding their implications for significant impact. As we expressed in our cover letter (excerpt pasted below), we believe that our results even in their current form represent substantive scientific progress.

Cover letter excerpt: "*Oscillatory dynamics play a major role in coordinating neural interactions. Many studies have examined the roles that synchronization and de-synchronization in specific frequency bands or specific pairs of frequency bands play in various behavioral functions. Rather than focusing on specific frequencies and functions, the goal of this study was to provide a big picture of intrinsic cross-frequency dynamics. Do cross-frequency dynamics have a simple intrinsic global structure? Do rapid and slow interactions have different structures? Having a global picture of intrinsic oscillatory activity would facilitate an understanding of how intrinsic dynamics are designed to accommodate the structures of sensory and behavioral dynamics.*

We systematically investigated power-based temporal associations between a broad range of oscillation frequencies both within and across EEG-based current sources (sites). Power-based associations are suitable

for providing a global picture of cross-frequency dynamics because (1) phase-based associations would be particularly impactful in the presence of power-based associations, (2) power-based associations at constant Δf would reveal global distributions of likely phase-power couplings, and (3) power-based associations can operate on characteristic timescales (independently of the interacting frequencies).

We have determined that large power fluctuations across all sites occur at two characteristic timescales, sub-seconds (within 500 ms) and seconds (~3.75 s). Importantly, we have discovered that the global patterns of spectral-power associations are distinct for the two timescales in a surprisingly simple manner. The sub-second-timescale associations are characterized by the diagonal, reflecting within-frequency associations, and lines parallel to the diagonal, reflecting associations at constant Δf's (implying power-phase coupling), whereas the seconds-timescale associations are characterized by columnar and square-shaped associations reflecting a broad range of frequencies being associated with a specific frequency band or with one another. Full functional implications of these simple structures await future investigations into how these intrinsic structures adjust to sensory dynamics and task demands. Nevertheless, one aspect of the current results supports a concrete interpretation. While recent studies have demonstrated the α-band rhythm of visual perception and its association with occipital α-band oscillations, our results suggest that occipital α-band oscillations also play a role in organizing higher-frequency oscillations into ~10 Hz amplitude-modulated packets to communicate with other areas."

2) A lot of spectral-power association plots are used to illustrate the sub-second and seconds timescale modulations within single site or cross-site. It is interesting that cross-frequency associations within-site represent the amplitude modulation with Δf and the cross-site associations centralized on the diagonal without Δf. These findings are interesting, but it is difficult for audiences to catch your findings if there is no quantitative statements.

A brief "take-home message" of our results would be, "the (fast) sub-second-timescale coordination of spectral power is limited to local amplitude modulation and insulated within-frequency long-distance interactions, whereas the characteristic columnar patterns of cross-frequency interactions emerge on the (slower) seconds timescale."

Although these are categorical (rather than quantitative) inferences, we think that they may still be useful to researchers studying computational modeling and physiological mechanisms because the current results may indirectly provide computational constraints. In the general discussion section, we also discuss semi-quantitative inferences: (1) the within-site and cross-site associations involving the posterior and other regions on the sub-second and seconds timescale consistently suggest that occipital processing communicates with other regions in 10 Hz amplitude-modulated packets, (2) the obtained patterns of spectral-power associations are quantitatively consistent with prior results on phase-amplitude coupling and feedback-feedforward interactions in terms of the implicated frequency bands, (3) our results have revealed some specific long-distance spectral-power associations that appear to defy the general trend of diminishing strengths of associations with longer inter-current-source distance, and (4) in response to a comment from Reviewer 1, we provide some evidence suggesting that the posterior Δf ~ 10 Hz and lateral Δf ~ 16 Hz associations on the sub-second timescale are quantitatively related to specific phase-amplitude coupling (please also see our response to Reviewer 1's comment #3). We agree that these inferences are not quantitative in the sense that they are not embedded within a coherent architecture of a computational model. As we apply the current spectral-power analysis method to EEG data from a variety of behavioral conditions, we hope to understand quantitative principles that predict how the distinct spectral-power associations on the sub-second and seconds timescales adjust to various perceptual and cognitive demands and how those adjustments relate to other local and global measures of cross-frequency interactions.

3) Only the cross-site association between one of the referenced sites (AFz, Oz, Cz, T7 & T8) and one out of the other electrodes were presented in this manuscript. There are a total 64*63/2 cross-site associations can be observed in the functional connectivity using 64-channel montage. However, it is impossible to illustrate all

cross-site associations in this manuscript. My opinion is to develop a quantitative measure for spectral-power association. The quantitative measure benefits to describing your findings and make it possible to conduct further analysis to functional connectivity for different conditions in future works.

We appreciate this comment. We did look at site pairs that are not presented in the paper (though we did not exhaustively examine all possible pairs). We have concluded that presenting the cross-frequency associations from all sites to representative reference sites would most effectively illustrate all of the notable patterns of associations.

Regarding the suggestion to develop a global statistic of spectral-power associations based on our results, others have used graph-theory metrics as summary statistics to describe global aspects of inter-region coupling per frequency band. While summary statistics can succinctly quantify global features, in our view, the goal of the current project was to analyze cross-frequency spectral-power associations with minimal transformations to facilitate direct comparisons between local (within-site) and cross-site interactions.

4) Only eye-closed state was investigated in this study, I am interested for what kind within-site and sub-second association (as shown in Figure 3) will be presented in compared with the outcome of eye-close condition. If it possible, authors can provide some statements to describe this.

As briefly mentioned at the end of the general discussion section, we have some preliminary results including the analyses applied to the EEG data collected while participants viewed a nature video, viewed various flickering stimuli, or engaged in various cognitive tasks. These preliminary results are reported in our pending grant proposals. When sufficient sample sizes are reached on these ongoing studies, we plan to publish the results to provide insights into how the intrinsic spectral-power associations on the fast and slow timescales adjust to perceptual and cognitive demands. Nevertheless, we feel that the current set of results characterizing intrinsic spectral-power association patterns will be useful for researchers who may wish to apply the analyses to conditions of their choosing. The current results per se also provide insights in that no prior results or theories predicted the distinct dependence of spectral-power associations on the sub-seconds and seconds timescales.

5) In the second paragraph of page 6 for generating phase-scrambled controls authors mentioned “we chose discrete cosign transform, DCT”. Is this statement correct? The full name of DCT should be “discrete cosine transform”, isn’t it?

Fixed. We thank the reviewer for detecting this typo.

It may seem that we tend to simply rebut Reviewer 2’s comments. We wish to clarify that we do understand Reviewer 2’s dissatisfaction with the paper. We share his/her view that the paper would have more impact if we included results from various behavioral conditions to elucidate how the intrinsic spectral-power associations on the fast (sub-seconds) and slow (seconds) timescales adjust to perceptual conditions and behavioral demands. We are working on these aspects, but it is likely to take significant time before we will have fully analyzed and understood the new results to draw substantive inferences regarding the potential physiological and functional implications of the intrinsic spectral-power associations we report in this paper. In addition, the manuscript is already quite long. For the reasons stated in the cover letter pasted above, we also feel that the current set of results will be useful (and potentially interesting) to the scientific community even without a fuller understanding of their physiological and functional significance. We feel that the revised manuscript with the substantially augmented scope of analyses (in response to the reviewers’ comments) is suitable for *PLoS ONE* especially as it focuses on disseminating rigorous and reliable scientific results even if their impact may be less clear.
